# Effects of Three Antecedents of Patient Compliance for Users of Peer-to-Peer Online Health Communities: Cross-Sectional Study

**DOI:** 10.2196/14006

**Published:** 2019-11-11

**Authors:** Anne-Françoise Audrain-Pontevia, Loick Menvielle, Myriam Ertz

**Affiliations:** 1 École des Sciences de la Gestion Université du Québec à Montréal Montréal, QC Canada; 2 École des Hautes Études Commerciales du Nord Nice France; 3 Université du Québec à Chicoutimi Chicoutimi, QC Canada

**Keywords:** online social networking, patient empowerment, patient compliance, patient satisfaction, structural equation modeling

## Abstract

**Background:**

Over the past 50 years, patient noncompliance has appeared as a major public health concern and focus of a great deal of research because it endangers patient recovery and imposes a considerable financial burden on health care systems. Meanwhile, online health communities (OHCs) are becoming more common and are commonly used by individuals with health problems, and they may have a role in facilitating compliance. Despite this growing popularity, little is known about patient compliance predictors for OHCs’ users.

**Objective:**

This study aimed to investigate the extent to which participating in OHCs may trigger higher levels of compliance. It identified 3 interrelated predictors that may affect patient compliance: patient empowerment gained through peer-to-peer OHCs, satisfaction with the physician, and commitment to the physician.

**Methods:**

A Web-based survey tested the conceptual model and assessed the effects of patient empowerment gained through OHCs on patient satisfaction and commitment to the physician, as well as the effects of these 3 predictors on patient compliance with the proposed treatment. Members of peer-to-peer OHCs were asked to answer an online questionnaire. A convenience sample of 420 patients experiencing chronic illness and using peer-to-peer OHCs was surveyed in August 2018 in Québec, Canada. A path analysis using structural equation modeling tested the proposed relationships between the predictors and their respective paths on patient compliance. The mediation effects of these predictor variables on patient compliance were estimated with the PROCESS macro in SPSS.

**Results:**

The findings indicated that patient empowerment gained through OHCs was positively related to patient commitment to the physician (beta=.69; *P*<.001) and patient compliance with the proposed treatment (beta=.35; *P*<.001). Patient commitment also positively influenced patient compliance (beta=.74; *P*<.001). Patient empowerment did not exert a significant influence on patient satisfaction with the physician (beta=.02; *P*=.76), and satisfaction did not affect compliance (beta=−.07; *P*=.05); however, patient satisfaction was positively related to patient commitment to the physician (beta=.14; *P*<.01). The impact of empowerment on compliance was partially mediated by commitment to the physician (beta=.32; 95% CI 0.22-0.44) but not by satisfaction.

**Conclusions:**

This study highlights the importance of peer-to-peer OHCs for two main reasons. The primary reason is that patient empowerment gained through peer-to-peer OHCs both directly and indirectly enhances patient compliance with the proposed treatment. The underlying mechanisms of these effects were shown. Second, commitment to the physician was found to play a more critical role than satisfaction with the physician in determining patient-physician relationship quality. Overall, our findings support the assumption that health care stakeholders should encourage the use of peer-to-peer OHCs to favor patient empowerment and patient commitment to the physician to increase patient compliance with the proposed treatment.

## Introduction

### Context

Defined as the extent to which a patient’s behavior coincides with the medical or health advice given by a health care specialist, patient compliance is of particularly critical importance for people with chronic health problems [1-3]. In developed countries, the World Health Organization [4] estimates that only 50% of chronically ill patients follow their prescribed treatment. Numerous empirical studies have aimed at describing and understanding patient compliance and corollary noncompliance over the past decades [5]. The literature emphasizes that patients’ lack of compliance with prescribed therapeutic regimens jeopardizes their health, adversely affects treatment outcomes, and leads to wasted health care resources [6]. Prior research has shown that nonadherence may cause 125,000 avoidable deaths each year and cost US $100 billion annually in preventable health care expenditures [7]. By seeking to unveil the factors associated with lack of compliance, scholars identified demographic and cultural differences as well as psychological or social factors, among others [8]. However, the literature highlights a dearth of consistencies and consensus regarding the determinants of patient compliance.

As increasing patient compliance is estimated to be more critical to improving the health of a population than any advancement in medical treatment [4,9], it is of strategic interest to understand the determinants of patient compliance, particularly in the era of the *medical internet*. Today’s digitization of health care holds promising perspectives for improving patient commitment and compliance [10]. Online health communities (OHCs) emphasize user-generated content and make it possible for users to exchange medical information anonymously, with no temporal or geographical constraints. OHCs are small virtual discussion groups in which people with a common concern about a health topic share information, experiences, and feelings; provide advice to fellow members; and provide social and emotional support [11,12]. Although these communities can present disadvantages, such as the spread of misinformation and unreliable support or advice, OHCs play a role in heightening patients’ sense of empowerment as they feel better informed and guided by relevant others [13]. Prior research has shown that communication between the physicians and patients in these communities enhances patient compliance [14]. However, despite the considerable development of peer-to-peer OHCs, there is a lack of evidence regarding their effects on patient compliance.

This study examined the relationships between 3 predictors that are theoretically and nomologically related to patient compliance, for patients who are active on peer-to-peer OHCs. These predictors were patient empowerment, patient satisfaction with their physician, and patient commitment to the relationship with the physician. We analyzed these constructs because they have been identified as critical predictors of patient compliance [15,16]. Previous research has shown that patient empowerment predicts patient commitment to the relationship with the physician [17]. In this study, we focused on patient compliance with recommended treatment, that is, the extent to which the patient adheres to prescriptions and treatment recommendations targeted to his or her disease. We intended to answer the following research questions: what are the antecedents of patient compliance with the treatment?, how does empowerment impact patient compliance with the treatment?, and what is the mechanism underlying the impact of empowerment on patient compliance with the treatment?. This study may equip physicians, other health care professionals, scholars, managers, and decision makers alike, with improved insights into a promising and original avenue for encouraging chronically ill patients to comply with their treatment.

### Theoretical Background and Model

Given the growing importance of a patient-centric perspective in contemporary health care, patient empowerment has been the subject of considerable attention from researchers over the past decades [18,19]. This is a consequence of the focus on a perspective that considers patients as consumers who also increasingly embrace the self-management of their disease [20,21]. Despite the amount of research dedicated to this construct in the health care research domain, there is a surprising lack of a consensual definition [22,23]. Most definitions agree that patient empowerment refers to the enhanced ability of patients to understand and influence their health [24]. Patient empowerment is frequently conceived of as a multidimensional and cognitive concept that includes different competencies and skills [25]. It refers to patients’ control over their illness and or treatment as well as to their ability to understand and participate in the consultation and contribute to the decision process based on the support brought by the physician [11,17,24].

There appears to be a broad consensus on the virtuous effects of empowerment [26]. Empowered patients tend to be more in control of their disease because it helps them to reduce uncertainty and to develop better strategies to cope with the disease [27]. Patient empowerment may also enhance patient commitment toward the physician [17] and patient satisfaction with the physician [28]. Besides, patient empowerment is identified as a prerequisite for efficient patient-physician relationships [29], and it increases patient compliance with physician-proposed treatment [17,30]. It appears that patient empowerment leads to higher levels of self-efficacy and self-management, which in turn result in better outcomes such as patient quality of life while reducing costs for health care systems. For all these reasons, patient empowerment has become a priority for health care systems dealing with chronic diseases [21,31].

**Figure 1:**
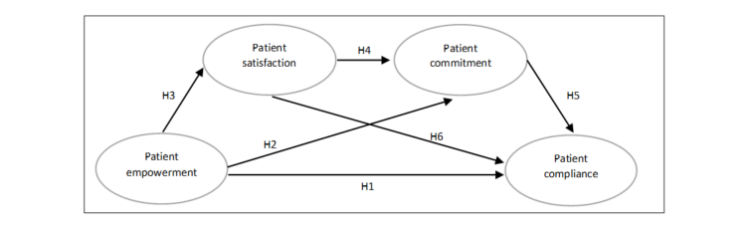
Theoretical model of the predictors of patient compliance showing hypothesized (H) relationships.

Building on previous research in the health care and consumer research fields [17,30,32], we proposed a conceptual model relating patient empowerment gained through peer-to-peer OHCs and patient compliance with physician-recommended treatment (Figure 1). 

Specifically, we hypothesized the following:

*H1: Patient empowerment gained through peer-to-peer OHC would positively relate to patient compliance with the proposed treatment.*

*H2: Patient empowerment gained through peer-to-peer OHC would positively relate to patient commitment to the physician.*

*H3: Patient empowerment gained through peer-to-peer OHC would positively relate to patient satisfaction toward the physician.*

In addition to the abovementioned direct relationships, we expected that the relationship between patient empowerment and patient compliance would be mediated by patient satisfaction with the physician and patient commitment toward the physician (Figure 1). These hypotheses relied on the relationship literature positing that both consumer satisfaction and commitment are prerequisites to a consumer-firm relationship. Hence, we hypothesized the following:

*H4: Patient satisfaction with the physician would positively relate to patient commitment toward the physician.*

*H5: Patient commitment toward the physician would positively relate to patient compliance with the proposed treatment.*

*H6: Patient satisfaction with the physician would positively relate to patient compliance with the proposed treatment.*

### Purpose and Contributions

Overall, 6 research hypotheses were tested on a total sample of 420 chronically ill OHC members by combining both structural equation modeling (SEM) and the bootstrapping-based PROCESS macro in SPSS (n=10,000 sample replications) to analyze the interrelationships within the proposed conceptual framework grounded in the fields of psychology, health care, and the social sciences. SEM has the particularity of testing and estimating simultaneously complex causal relationships between latent variables that are operationalized by manifest variables. Besides, SEM estimates random errors in the observed variables, thus reinforcing estimation accuracy. To assess the existence of mediating effects, we used the PROCESS macro in SPSS. This research contributes to the existing literature in several ways. First, it proposed a conceptual framework relating patient empowerment gained through peer-to-peer OHCs to patient compliance. Second, it tested both direct and indirect effects within patient empowerment gained through peer-to-peer OHCs in a framework of nomologically related variables. As such, the study highlighted the key contributors to patient compliance for chronically ill patients involved in peer-to-peer OHCs. Third, through the study’s focus on chronically ill patients, this research provides valuable insights to better support a population that is most vulnerable to death and disability worldwide [21,33].

## Methods

### Participant Recruitment and Data Collection

Data for this cross-sectional study were collected over a 3-week period in August 2018 from OHCs in Canada. A self-reported questionnaire was administered on the Web-based survey platform Qualtrics. The link to the questionnaire was posted on Canadian OHCs dedicated to chronic diseases for a representative sample from the Qualtrics panel using preset quotas. We focused on French-speaking peer-to-peer OHCs on which members report suffering from at least one chronic sickness such as, for example, cancer, diabetes, or obesity. Participants had to have visited an OHC at least once during the 3 months before the survey. The questionnaire was pretested (10 respondents) and pilot-tested (32 respondents) because of the translation and adaptation of items to French and to confirm validity and reliability. Informed consent was obtained from each participant before starting the survey. Participation was voluntary and anonymous. An ethics committee has validated and approved the research protocol.

### Sample Size

The study size was estimated based on a rule of thumb of roughly 10 respondents per item in the study [34]. With a total of 14 items, a minimum sample size of 140 observations was required. An initial pool of 1760 respondents entered the survey; 420 of these respondents matched the inclusion criteria. After removing incomplete or invalid questionnaires, the final operative sample was composed of 315 observations.

## Results

### Characteristics of the Study Population

Demographic characteristics of the sample are shown in Table 1. Of the 315 respondents, 144 (45.7%) were female and 134 (42.5%) were younger than 35 years. In addition, 208 out of 305 (68.2%) of the respondents had a university degree. Within this chronically ill patient sample, 132 out of 306 (43.1%) respondents suffered from type 1 or type 2 diabetes; thus, diabetes was the most represented pathology. Furthermore, 66 out of 306 (21.6%) respondents declared that they suffered from obesity. Regarding the duration of the illness, most of the respondents, 236 out of 315 (75%), declared that they had suffered from a chronic illness for at least 1 year.

**Table 1:** Demographic characteristics of study participants (N=315).

Demographic characteristics		Value, n (%)
**Gender**		
	Female	144 (45.7)
	Male	171 (54.3)
**Age (years)**		
	Less than 18	6 (1.9)
	18-24	45 (14.3)
	25-34	70 (22.2)
	35-49	110 (34.9)
	50-65	73 (23.2)
	More than 65	11 (3.5)
**Education**		
	No education	30 (9.5)
	Secondary	67 (21.3)
	Undergraduate	84 (26.7)
	Graduate	49 (15.6)
	Postgraduate	75 (23.8)
	Missing data	10 (3.1)
**Chronic disease**		
	Diabetes type 1	62 (19.7)
	Diabetes type 2	70 (22.2)
	Obesity	66 (21.6)
	HIV	3 (1)
	Cancer	15 (4.7)
	Other diseases	90 (28.5)
	Missing data	9 (2.9)
**Duration of the illness (years)**		
	<1	78 (24.8)
	1 to <2	138 (43.8)
	2 to <3	64 (20.3)
	>3	34 (10.8)
	Missing data	1 (0.3)

### Measurement of Variables

As our research investigated OHCs from consumer behavior and marketing perspectives, we have grounded our measurements in both the behavioral and psychological theories as well as measurement tools. For the measure of empowerment, we used an adapted version of Ouschan et al’s validated scale [17], consisting of 15 items, reduced to 4 items (mean 3.28, SD 1.58) after the factor analysis revealed several inconsistencies with this scale (see details in the next subsection). We measured patient satisfaction by adapting Oliver’s scale [35], consisting of 3 items (mean 3.83, SD 1.79). Patient commitment was measured by 4 items from Morgan and Hunt’s commitment scale (mean 3.67, SD 1.91) [36]. Finally, patient compliance, our dependent variable, was captured with 3 items from Prigge et al’s compliance scale (mean 3.10, SD 1.62) [16]. All items were scored on 7-point Likert scales, ranging from 1 (*totally disagree*) to 7 (*totally agree*). The items retained at the end of the factorial analysis purification procedure are shown in Table 2. We used gender, age, nationality, province or country of residence, education, and occupation as control variables. To reduce measurement context effects and common method bias (CMB), the measurement items were randomized within the research questionnaire.

### Measurement of the Research Model

A set of preliminary analyses such as outliers, nonnormality checks, and descriptive data analysis was carried out. To check for CMB inherent to cross-sectional survey-based studies, Harman single factor test was run by performing factor analysis. The results revealed that the first factor does not explain more than 50% of the overall variance, which showed that CMB was not a concern. To test the measurement model, an exploratory factor analysis with the principal component analysis extraction method and the varimax rotation technique was carried out. It showed that the measurement structure explained 83.8% of the variance and that each item loaded significantly on its intended factor.

To assess the fit of the measurement model, a confirmatory factor analysis (AMOS in SPSS) was performed with the maximum likelihood estimation procedure, a robust method in latent variable modeling [37]. Different goodness-of-fit indices were used to estimate the quality of the model, namely, comparative fit index (CFI), normed fit index (NFI), nonnormed fit index (NNFI) greater than or equal to 0.950, standardized root mean residual (SRMR) less than or equal to 0.080, and root mean square error of approximation (RMSEA) less than or equal to 0.050 [38]. However, the fit of the model was consistently poor because of the empowerment items. This is not surprising as there is a lack of consensus on both the conceptualization and the measurement of empowerment [39,40]. According to the Construct definition, Object classification, Attribute classification, Rater identification, Scale formation, and Enumeration and reporting methodology, the construct appears thus misspecified, and the measurement tools supposed to measure this concept might actually measure something else [41]. Therefore, after several iterations, a total of 11 items were deleted from the empowerment scale. The resulting measurement model displayed good overall fit (*χ*^2^_36_=43.5 CFI=0.997, NFI=0.983, NNFI=0.995, SRMR=0.026, and RMSEA=0.028). Besides, all item loadings were significant and above the 0.70 threshold, and, as shown in Table 2, the average variance extracted (AVE) of each construct (from 0.644 to 0.799) was above the 0.50 threshold. Conjointly, these results demonstrate convergent validity [38]. As further shown in Table 3, the coefficient of reliability was higher than the AVE for each construct, thus reinforcing convergent validity [42].

**Table 2:** Constructs, items, means, standard loading, and standard deviation.

Construct and item		Standard loading	Mean (SD)
**Empowerment (physician support)**			
	When addressing my condition, my doctor focuses on health promotion	0.69	3.21 (1.57)
	My doctor provides clear instructions on what to do in different situations	0.78	3.27 (1.51)
	When appropriate, my doctor provides me with a written plan on how to control my chronic illness condition	0.82	3.33 (1.61)
	My doctor keeps me up to date with the most recent information on chronic illness conditions	0.83	3.32 (1.60)
**Commitment**			
	The relationship with my physician is important for me	0.85	3.00 (1.59)
	The relationship with my physician is something that I want to maintain	0.87	3.08 (1.50)
	The relationship with my physician is particularly important for me	0.77	3.25 (1.59)
**Satisfaction**			
	I am satisfied with my physician	0.92	3.67 (1.91)
	I think I made the good choice to choose my physician	0.93	3.90 (1.78)
	If I had to choose a physician, I would choose another one (reversed-polarity item)	0.92	3.92 (1.69)
**Compliance**			
	I take the medication prescribed by my doctor at the right time	0.62	3.40 (1.67)
	I take the right dosage of the medication prescribed by my doctor	0.76	2.84 (1.64)
	I follow the prescribed treatment regularly and continuously	0.66	3.06 (1.56)

**Table 3:** Psychometric properties.

Construct	Cronbach alpha	Average variance extracted	Average shared variance	Composite reliability	Empowerment	Satisfaction	Commitment	Compliance
Empowerment	.883	0.719	0.408	0.885	0.848	0.001	0.719	0.840
Satisfaction	.924	0.799	0.007	0.923	0.001	*0.894^a^*	0.139	0.030
Commitment	.895	0.741	0.425	0.895	0.719	0.139	*0.861*	0.859
Compliance	.779	0.644	0.481	0.783	0.840	0.030	0.859	*0.802*

^a^The italicized values in the diagonal refer to the average variance extracted (AVE) for each construct.

Pearson product-moment correlation analyses revealed both univariate and bivariate links between the variables. As suggested by Fornell and Larcker, each latent variable accounted for more variance, as shown in its AVE, than its shares with the other constructs in the model, as shown in the interconstruct correlations, except for compliance [42]. Although both correlations were below the maximum tolerable threshold of 0.90 for a correlation, we checked that compliance was unrelated to both empowerment (*r*=0.84) and commitment (*r*=0.85) at the .01 level, for which high correlations were found [43]. We constructed a confidence interval for both correlations (ie, correlation ± 1.96 x standard error [44]. If |1| is included in this interval, this indicates a lack of discriminant validity [44]. The resulting confidence intervals for the empowerment, compliance correlation (0.84 ± 1.96 x standard error 0.06; CI 0.72-0.95) as well as for the compliance, commitment correlation (0.85 ± 1.96 x standard error 0.06; CI 0.73-0.98) both excluded |1|, confirming discriminant validity. Both the coefficients of reliability (from 0.885 to 0.923) and Cronbach alpha (from .883 to .924) were high, indicating good construct reliability [42].

### Structural Model

**Figure 2:**
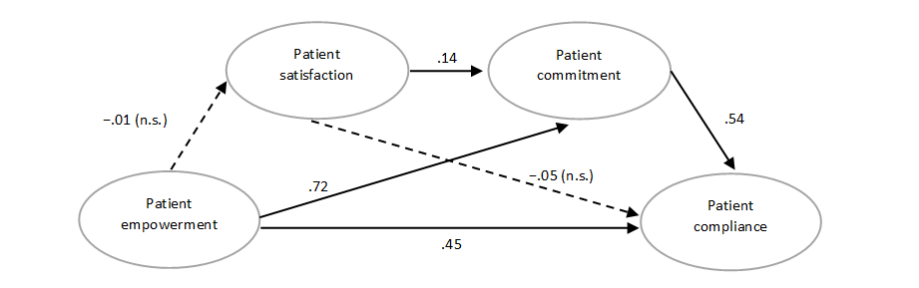
Structural model with results. ns: not significant.

We evaluated the fit of our structural model. The results suggest that the collected data adequately fit our research model as an appropriate model fit has been reached (*χ*^2^_35_=43.5; CFI=0.997, NFI=0.983, NNFI=0.995, SRMR=0.026, and RMSEA=0.028). The path results are visually summarized in Figure 2.

Overall, the results show that all the anticipated relationships of our model were supported except for the impact of empowerment on satisfaction and satisfaction on compliance. Empowerment was positively related to compliance, lending support to hypothesis 1 but suggesting a partial mediation effect of patient satisfaction and patient commitment on the relationship between patient empowerment and patient compliance. Empowerment positively influenced patient commitment, supporting hypothesis 2. However, patient satisfaction did not appear to play a significant role in explaining the effect of empowerment gained through OHCs and patient compliance with the prescribed treatment, as the relationship between patient empowerment and satisfaction was nonsignificant, as was the link between patient satisfaction and patient compliance. These results invalidate hypotheses 3 and 6, respectively. These findings suggest that the primary explanatory variable in the relationship was patient commitment as patient empowerment significantly increased the level of patient commitment. In turn, commitment improves compliance with the prescribed treatment. Collectively, these findings support hypotheses 2 and 5. The results provide preliminary evidence that patient commitment partially explains the relationship between empowerment gained through OHCs and enhanced patient compliance, whereas satisfaction does not (see Table 4).

**Table 4:** Standardized coefficients.

Estimated paths	Coefficient	SE	*t* test (*df*)	*P* value
Empowerment-Satisfaction	−0.01	0.09	−0.01 (35)	.76
Empowerment-Commitment	0.72	0.07	10.63 (35)	<.001
Satisfaction-Commitment	0.14	0.05	2.76 (35)	.005
Empowerment-Compliance	0.45	0.10	4.42 (35)	<.001
Satisfaction-Compliance	−0.05	0.04	−1.14 (35)	.05
Commitment-Compliance	0.54	0.10	5.28 (35)	<.001

### Bootstrapping Model

**Figure 3:**
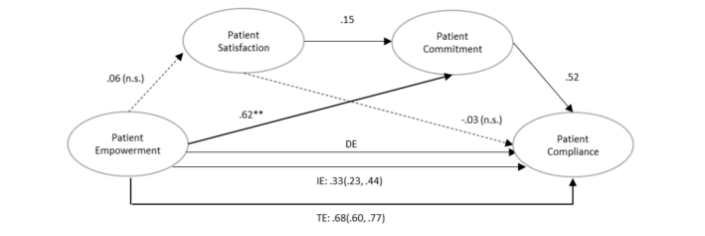
Research model with bootstrapping with direct, indirect and total effects. DE: direct effect; IE: indirect effect; TE: total effect; ns: not significant.

A serial mediation analysis, also called multiple-step multiple mediation [45], using the bootstrapping SPSS PROCESS macro in SPSS (model 6) [46] on 10,000 resamples, gave a more precise estimate of the existence, polarity, and magnitude of the mediation effect of commitment and cross-validated the nonsignificance of satisfaction in the overall model. In the PROCESS macro in SPSS, the indirect mediation effect is considered to be significant when the confidence interval of the regression coefficient does not include 0 (Figure 3).

The bootstrapping results replicated the SEM findings; all the relationships were significant except that of empowerment on satisfaction (beta=.05; *P*=.33) and satisfaction on compliance (beta=−.03; *P*=.39). These findings are consistent with the SEM results in AMOS in SPSS. Also in line with the SEM procedure, empowerment affected commitment (beta=.62; *P*<.001) and satisfaction positively influenced commitment (beta=.15; *P*<.01), whereas commitment influenced compliance (beta=.51; *P*<.001). The direct effect of empowerment on compliance was significant (beta=.35; *P*<.001) as was the indirect effect (beta=.32; 95% CI 0.22-0.44), both making up for a highly significant total effect (beta=.68; *P*<.001). Although unrelated to empowerment, satisfaction slightly improved patient compliance indirectly through heightened commitment. These results suggest that commitment only partially explains the effect of empowerment on compliance [45]. In other words, it is through the direct effect of patient empowerment as well as the enhanced patient commitment triggered by patient empowerment that patient compliance grows.

## Discussion

### Principal Findings

This study investigated the direct and indirect influence of the empowerment perceived by patients of OHCs—specialized in chronic illnesses—on compliance with recommended treatment. We examined this effect via a mediational model linking empowerment to patient satisfaction and patient commitment in a multiple-step multiple mediation model.

This research relied on the use of a confirmatory approach that enables the simultaneous estimation and testing of several relationships among the predictive variables of empowerment, commitment, and satisfaction and their effects on compliance. To our knowledge, this is the first study to address the effects of patient empowerment gained through peer-to-peer OHCs on patient compliance with the proposed treatment. As such, this study extends prior research [14] showing that communication between physicians and patients in OHCs positively affects patient compliance. Lu and Zhang [14] found that when patients interact with physicians in OHCs, they have a better assessment of the quality of internet health information and they have better preferences during decision-making processes. Those results also underlined that patient-physician concordance is enhanced. These mechanisms, then in turn, positively impact patient compliance. In line with these findings, our research specific to peer-to-peer OHCs confirms that these communities can be used as a powerful tool to enhance patient compliance. This study also complements past research [13] showing that exchanging information with professional moderators in OHCs increases patient empowerment and improves cooperation between the patient and the physician. It also extends previous research findings [24,25,47,48] highlighting that internet services contribute to enhance patient empowerment while demonstrating the underlying mechanism of how empowerment gained in OHCs enhances patient commitment toward the physician and patient compliance with the recommended treatment.

We used SEM not only because it allows for the testing of causal relationships among a set of variables within a hypothetico-deductive approach and because it estimates the strength of these relationships but also because it appraises measurement error. We also used the model 6 of the PROCESS macro in SPSS [45], on 10,000 resamples, to test the mediatory effects among the explanatory variables (Figure 3). Given the interrelationships that we hypothesized in our model (Figure 1), it was important to understand how such effects operate and whether some variables mediate the effect of other variables on the dependent variable, namely, patient compliance. Together, the SEM and Hayes PROCESS macro model approach underlined that 4 out of the 6 estimated paths of the research model were confirmed. Findings from this study highlight that out of the 3 antecedents of chronically ill patients’ compliance that we model, 2 of them, namely, patient empowerment and commitment, had a direct and strong effect on the intended dependent variable of compliance. In other words, this study reveals that patient empowerment and patient commitment to the relationship with the physician have strong positive direct effects on chronically ill patients’ compliance with recommended treatment, whereas patient satisfaction with the physician has no direct effect, but a mediating effect through patient commitment to the relationship with the physician, on compliance. Our data also highlight that patients’ heightened sense of empowerment affects patient compliance not only directly but also indirectly by exerting a strong positive effect on patient commitment.

Prior research has shown that patient empowerment gained through peer-to-peer OHCs is determined by both the information utility found and shared in the communities and psychological benefits such as emotional support [49]. In the same vein, the computer-mediated social support gained through OHCs was found to positively alter the patient’s commitment toward the physician [50]. Therefore, it is of strategic importance to posit both medical information sharing and social support as the core of the peer-to-peer OHC design. Specifically, patients should be encouraged to participate in peer-to-peer OHCs. As Johnston et al [49] underlined it, participation determines information utility and social support. Therefore, while designing OHCs communities, managers of those platforms should focus on implementing mechanisms that support the active participation of their members to elicit information sharing and enhance social support. Though still in development, the literature provides managers with useful guidelines about ways to stimulate such forms of participation and engagement. As participation in OHCs is intertwined with the perceived quality of the information shared [49], a *bottom-up* approach is advised to encourage self-regulation and self-rating processes of the information in these health support communities [51]. Third parties could evaluate the information shared, and there could be enforcement mechanisms in case of fraudulent or harmful information [51]. These mechanisms are expected to enhance the quality of the information shared and consequently patient’s participation, which in turn will affect both the perceived information and perceived social support. Another avenue consists of providing tools to educate community users, to promote health literacy, and to help users feel confident to engage with those communities.

Interestingly, patient satisfaction with the physician did not appear to have a direct effect on patient compliance and was not determined by patient empowerment gained through OHCs (Figure 3).

Past research emphasized the emergence of dysfunctional empowerment emerging from OHCs, in that people with support from these communities may become less invested in their relationships with their physicians (eg, distrust of the physician, feelings of superiority over professional knowledge, and overconfidence in relation to the physician) [13]. Yet, our study shows that OHCs might also contribute to patients’ compliance with prescribed treatment. The study underlines that satisfaction with the physician is not a significant contributor to the process, thus suggesting the occurrence of potential dysfunctional empowerment [13] that materializes in patient dissatisfaction. However, we did not control for this effect in the study, so it is difficult to estimate to what extent the low significance of satisfaction is related to dysfunctional empowerment. Importantly, the absence of influence of satisfaction does not prevent empowerment from exerting a significant effect on patients both directly and indirectly, suggesting that if dysfunctional empowerment is there, its effect is minimal in comparison with the overall positive influence of empowerment on compliance. This claim will need to be better substantiated by future research.

### Practical Implications

Results from our study have several implications for health care services. First, they suggest that health care stakeholders should aim at enhancing patient empowerment on peer-to-peer OHCs. These communities provide their members with both informational and emotional support, enabling them to reduce uncertainty and make critical decisions about their health [48,52]. Defined as the ability to shape the composition of one’s choice, patient empowerment positively influences patients’ ability to make their own decisions about their health care [53]. Today, peer-to-peer OHCs contribute to the transfer of power and mastery from physicians to patients and favor improved health behaviors, better health outcomes, and reduced health care costs [11]. This study suggests that when empowered, peer-to-peer OHC members with a chronic disease report better compliance with their recommended treatments. It should be noted that this probably requires patients to have a reasonable level of literacy. Patients who do not have the technical health knowledge may not be able to join these communities. In the same vein, this suggests that patients are confident and competent enough to use peer-to-peer OHCs, which is not always the case. Therefore, efforts should be made to educate patients to overcome these shortcomings and avoid inadvertent exclusion of segment population. Knowing that half of all adults worldwide have a chronic condition [3] and that 86% of internet users living with a chronic condition have searched online for medical information in 2007 [53], our findings are of considerable interest for both physician and health care systems. Our results indicate that peer-to-peer OHCs are powerful social tools that enhance chronically ill patients’ commitment to the physician and compliance. This is why health care systems and physicians should promote peer-to-peer OHCs among patients suffering from a chronic condition.

Ultimately, this could be achieved by integrating these platforms in health care systems and physicians’ workflows. However, research is needed to indicate how these communities could be integrated; we believe that patients could be invited to join these communities at an early stage of their relationship with the physician. This would probably require some time and effort from the physician or other health care workers to accompany the patient in his/her joining of these communities. This investment could prove to be beneficial in the mid- and long-run in the patient-physician relationship, bearing in mind that these communities also enhance disease self-care and reduce health care utilization [13]. While improving the workflow of physicians, these technological tools could contribute to a much broader phenomenon colloquially denominated as *shadow work* [54]. OHCs increase patients’ workload of searching and analyzing vast quantities of information originating from patient exchange online, where previously the physician acted as the main filter and interpreter of medical information. The performance of this *shadow work* by the patient may decrease the physician’s value in the eye of the patient. However, physicians remain central figures in the health care process, and with the continuous growth of these OHCs, doctors should act as information guides by helping patients to navigate through the complex net of information available to them online. This will make patients’ information searches easier and more productive.

Overall, these results stress the importance of empowering patients and increasing commitment to the relationship that they have with their physician, and importantly, the possibility of using peer-to-peer OHCs as a means to do so. As peer-to-peer OHCs can help their members to gain more autonomy and efficacy in the self-management of their chronic disease, they positively influence users’ well-being. As these communities reduce health care utilization, they contribute to a better use of health care resources. In that sense, our findings concur with those of Joglekar et al [55]. As OHCs provide the positive outcomes highlighted in this study and as they constitute a major source of health information for patients [56], health care stakeholders should encourage patients with chronic conditions to use peer-to-peer OHCs.

### Limitations of This Study

Though this study highlights the implications of empowerment gained through peer-to-peer OHCs for compliance, we identified several limitations to the generalization of the results of this study. First, regarding the measurement of the empowerment construct, only 1 dimension, referring to physician support, was identified in our sample. It should also be noted that although we aimed at limiting the bias inherent to self-reported surveys, there remains potential bias, such as social desirability and selective recall [57]. Second, our model did not include all the predictors that have been reported in previous research so far. We focused on a key set of predictors, especially empowerment, because the literature has emphasized that this construct had a strong influence on patient compliance [58]. Prior research highlights that OHCs provide their members with the social support that leads them to feel a heightened sense of empowerment [59,60]. Other related constructs might be relevant as well, such as self-efficacy or perceived usefulness. Third, our SEM model conceptualized patient compliance and its predictors in a mediational analysis. However, some variables may moderate the relationships that we studied. In particular, we believe that patient literacy, and more specifically, electronic health (eHealth) literacy [61], moderates these relationships; it would be of interest to investigate its moderating effect on the paths we identified. Fourth, participants in our sample were French-speaking patients with chronic illness in the Province of Québec, Canada.

### Directions of Future Research

To overcome the limitations mentioned in the previous section, future research should focus on the following specific aspects. First, further research should investigate the effect of the construct of social support gained through OHCs to increase our understanding of the effect of OHCs on chronically ill patients’ compliance. Second, the moderating impact of patient literacy or eHealth literacy should be put to empirical test. Third, replication studies are needed to test the proposed theoretical framework in other provinces and countries with different health care systems. The external validity of the findings would increase if they can be replicated across various other medical care systems, contexts, and countries. Indeed, the Province of Québec, similar to Canada, offers a universal health care system to its citizens. While providing richer and more idiosyncratic insights into the impact of empowerment on compliance, it would be of interest to test our model in other types of health care systems that are entirely private or have a hybrid configuration. Fourth, future studies relying on qualitative methods, such as interviews or focus groups, would bring meaningful insights while limiting the bias inherent to self-reported studies.

### Conclusions

The findings indicate that patient empowerment and commitment to the relationship with the physician are the 2 key predictors that enhance patient compliance with the prescribed treatment. Interestingly, patient satisfaction with the physician is found to impact patient compliance but through the mediating effect of patient commitment. Though patient empowerment has been shown to be critical in the health care literature, to the best of our knowledge, little research has empirically estimated how empowerment gained through peer-to-peer OHCs affected patients’ propensity to comply with the prescribed treatment. This study suggests that health care stakeholders should encourage the use of peer-to-peer OHCs to enhance patient commitment and, ultimately, patient compliance with the physician. We believe that this research stream is and shall continue to be of considerable interest as compliance is a major public health concern impacting the costs and performance of health care systems.

## Abbreviations

AVE: average variance extracted
CFI: comparative fit index
CMB: common method bias
eHealth: electronic health
FODAR: Fonds de développement académique du réseau
NFI: normed fit index
NNFI: nonnormed fit index
OHC: online health community
RMSEA: root mean square error of approximation
SEM: structural equation modeling
SRMR: standardized root mean residual
